# Supplementary material for: The timing and asymmetry of plant–pathogen–insect interactions
Source: Proc Biol Sci. 2020 Sep 23;287(1935):20201303. doi: 10.1098/rspb.2020.1303 (PMC7542815; doi:10.1098/rspb.2020.1303)
Supplement: Table S1. [file rspb20201303supp1.docx]

**Table S1.** Models fitted for plant performance. [F] = Fixed effects, [R] = Random effects. Performance data from week 4 onwards were included in the models; at this time, all attackers were present on the plants for at least 1 week. Date was treated as a factor in the model, and acorn size as a continuous factor. N = 300 replicates (plants) in each model.

| Question | Response variables | Transformation | Models |
| --- | --- | --- | --- |
| Do attackers impact plant performance? | *Plant height*  *Number of developed leaves*  *Leaf size*  *Number of shoots* | sqrt  sqrt  sqrt  log | ~ treatment [F] + date [F] + treatment × date [F] + treeID [R] + acorn size [F] + acorn size × treatment [F] |
