# Supplementary material for: The timing and asymmetry of plant–pathogen–insect interactions
Source: Proc Biol Sci. 2020 Sep 23;287(1935):20201303. doi: 10.1098/rspb.2020.1303 (PMC7542815; doi:10.1098/rspb.2020.1303)
Supplement: Table S2. [file rspb20201303supp2.docx]

**Table S2**. Overview of the additive risk model, used to explore the effects of attacker identity and additive vs. non-additive effects of attacker combinations on plant performance (height, number of developed leaves, leaf size and the number of shoots; all log-transformed). Treatments included in this model were: 1, 2, 3, 4, 5, 6 and 7 (that is, healthy seedlings, seedlings with one attacker or seedlings with two co-occurring attackers), n = 140 plants per model, 20 per treatment. For the covariates: [F] = Fixed effects, [R] = Random effects.

| Questions | Hypotheses | Model |
| --- | --- | --- |
| Do attackers impact plant performance? | *Plant performance is expected to be negatively impacted by attackers, with impact intensity depending on attacker identity.* | Plant performance ~ Mildew + Aphids + Caterpillar + date  + Mildew × Aphids + Mildew × Caterpillar + Aphids × Caterpillar  + Mildew × date + Aphids × date + Caterpillar × date  + Mildew × Aphids × date + Mildew × Caterpillar × date + Aphids × Caterpillar × date  + TreeID [R] + Acorn size [F] |
| Are the effects of co-occurring attackers on plant performance additive, synergistic or antagonistic? | *As based on a trade-off between SA and JA, the effects of co-occurring attackers vs. single attackers on plant performance are expected to be: Mildew and aphids: antagonistic*  *Mildew and caterpillar: synergistic*  *Aphids and caterpillar: synergistic* |  |
