# Supplementary material for: The timing and asymmetry of plant–pathogen–insect interactions
Source: Proc Biol Sci. 2020 Sep 23;287(1935):20201303. doi: 10.1098/rspb.2020.1303 (PMC7542815; doi:10.1098/rspb.2020.1303)
Supplement: Table S3. [file rspb20201303supp3.docx]

**Table S3**. Overview of fitted repeated measures models for aphid and mildew performance. Ap = aphids, M = powdery mildew, C = caterpillar. Response variables for aphids and mildew were log-transformed, and caterpillar growth rate was log-transformed (as well as starting weight), though the other response variables for caterpillars were not transformed. Date was treated as a factor in the models. [F] = Fixed effects, [R] = Random effects.

| **Questions** | **Response variables** | **N** | **Model type** | **Treatment comparisons** | **Models** |
| --- | --- | --- | --- | --- | --- |
| Do attackers impact one another’s performance? | *M coverage (%)* | 60 | lmer  lmer | 2, 5, 9  2, 6, 11 | ~ treatment [F] + date [F] + treatment × date [F] + treeID [R] |
|  | *Ap population size* | 60 | lmer | 3, 5, 8  3, 7, 13 |  |
|  | *C growth rate*  *C final weight*  *C pupal weight*  *C development time*  *C survival* | 60  81 (mildew)  130 (healthy) | lm  lm  lm  lm  glm (family = binomial) | 4, 7, 12  Mildew vs. healthy diet | ~ treatment [F] + starting weight [F]  ~ treatment [F] + starting weight [F]  ~ treatment [F] |
| Do early arriving attackers influence interaction outcome of later arriving attackers? | *M coverage (%)* | 40 | lmer  lmer | 5, 14  6, 15 | ~ treatment [F] + date [F] + treatment × date [F] + treeID [R] |
|  | *Ap population size* | 40 | lmer | 5, 14 |  |
