# Supplementary material for: The timing and asymmetry of plant–pathogen–insect interactions
Source: Proc Biol Sci. 2020 Sep 23;287(1935):20201303. doi: 10.1098/rspb.2020.1303 (PMC7542815; doi:10.1098/rspb.2020.1303)
Supplement: Table S4. [file rspb20201303supp4.docx]

**Table S4**. Questions and hypotheses for the specific treatment comparisons included in the fitted models for aphid and mildew performance, and for the effect of diet on caterpillar performance (see *Table S3*). Ap = aphids, M = powdery mildew, C = caterpillar.

| Treatment comparison | Questions | Hypotheses |
| --- | --- | --- |
| 3, 5, 8 | Difference in Ap performance when being alone compared to co-occurring with M?  And is A performance differentially impacted when M arrives earlier than Ap? | *Ap performance lower when co-occurring with M*  *Ap performance lower when M arrives earlier* |
| 3, 7, 13 | Difference in Ap performance when being alone compared to co-occurring with C?  And is A performance differentially impacted when C arrives earlier than Ap? | *Ap performance higher when co-occurring with C*  *Ap performance higher when C arrives earlier* |
| 5, 14 | Is the performance of Ap (co-occurring with M) altered when C arrives earlier on the plant? | *Ap performance higher when C arrives earlier* |
| 2, 5, 9 | Difference in M performance when being alone compared to co-occurring with Ap?  And is M performance differentially impacted when Ap arrive earlier than M? | *M performance lower when co-occurring with Ap*  *M performance lower when Ap arrives earlier* |
| 2, 6, 11 | Difference in M performance when being alone compared to co-occurring with C?  And is M performance differentially impacted when C arrive earlier than M? | *M performance higher when co-occurring with C*  *M performance higher when C arrives earlier* |
| 4, 7, 12 | Difference in C performance when being alone compared to co-occurring with Ap? And is C performance differentially impacted when Ap arrive earlier than C? | *C performance higher when co-occurring with A*  *C performance higher when A arrives earlier* |
| Mildew vs. healthy diet | Is the performance of C altered when feeding on mildew-infected vs. healthy leaves? | *C performance higher when feeding on M leaves* |
| 5, 14  6, 15 | Is the performance of M, co-occurring with Ap or C, altered when C or Ap arrives earlier on the plant? | *M performance higher when C arrives earlier*  *M performance lower when Ap arrives earlier* |
| 5, 14 | Is the performance of Ap, co-occurring with M, altered when C arrives earlier on the plant? | *A performance is higher when C arrives earlier* |
