# Supplementary material for: The timing and asymmetry of plant–pathogen–insect interactions
Source: Proc Biol Sci. 2020 Sep 23;287(1935):20201303. doi: 10.1098/rspb.2020.1303 (PMC7542815; doi:10.1098/rspb.2020.1303)
Supplement: Table S5. [file rspb20201303supp5.docx]

**Table S5**. The effect of treatment, date, treatment × date and acorn size on plant performance. Shown are the results from the repeated measures models specified in *Table S1*. Acorn size was included as a covariate in the model. Significant values are indicated in bold. N = 300 plants, with 20 plants per treatment. Shown are degrees of freedom, test statistics and p-values.

| Response variables | Treatment | | | Date | | | Treatment × Date | | | Acorn size | | | Acorn size × Treatment | | |
| --- | --- | --- | --- | --- | --- | --- | --- | --- | --- | --- | --- | --- | --- | --- | --- |
|  | **DF** | **Χ^2^** | **p-value** | **DF** | **Χ^2^** | **p-value** | **DF** | **Χ^2^** | **p-value** | **DF** | **Χ^2^** | **p-value** | **DF** | **Χ^2^** | **p-value** |
| Plant height | 14 | 11.8 | 0.62 | 5 | 1328.0 | **<0.001** | 70 | 163.9 | **<0.001** | 1 | 96.4 | **<0.001** | 14 | 8.7 | 0.85 |
| Number of developed leaves | 14 | 18.3 | 0.20 | 5 | 792.4 | **<0.001** | 70 | 278.0 | **<0.001** | 1 | 80.0 | **<0.001** | 14 | 7.9 | 0.90 |
| Leaf size | 14 | 29.4 | **0.01** | 5 | 213.9 | **<0.001** | 70 | 193.7 | **<0.001** | 1 | 73.2 | **<0.001** | 14 | 17.5 | 0.23 |
| Number of shoots | 14 | 10.9 | 0.69 | 5 | 1071.8 | **<0.001** | 70 | 130.1 | **<0.001** | 1 | 24.6 | **<0.001** | 14 | 4.0 | 1.00 |
