# Supplementary material for: The timing and asymmetry of plant–pathogen–insect interactions
Source: Proc Biol Sci. 2020 Sep 23;287(1935):20201303. doi: 10.1098/rspb.2020.1303 (PMC7542815; doi:10.1098/rspb.2020.1303)
Supplement: Table S6. [file rspb20201303supp6.docx]

**Table S6.** The impact of attackers and attacker combinations on plant performance. A) Shown are the results from the additive risk model specified in *Table S2*, with i) the effect of individual attackers on plant performance; ii) additive effects of co-occurring attackers on plant performance; iii) variation through time of the effect of individual attackers on plant performance; iv) variation through time of additive effects of co-occurring attackers on plant performance; and v) the effect of acorn size and date. Significant values are indicated in bold. N = 140 plants; 20 healthy, 20 with mildew, 20 with aphids, 20 with caterpillars and 60 plants with two co-occurring attackers. Shown are test statistics and p-values.

|  |  | **Height** | | **Developed leaves** | | **Leaf size** | | **Shoots** | |
| --- | --- | --- | --- | --- | --- | --- | --- | --- | --- |
|  |  | **Χ^2^** | **p-value** | **Χ^2^** | **p-value** | **Χ^2^** | **p-value** | **Χ^2^** | **p-value** |
| i) | Mildew | 0.09 | 0.77 | 1.72 | 0.19 | 0.17 | 0.68 | 0.52 | 0.47 |
|  | Aphid | 0.10 | 0.75 | 0.08 | 0.78 | 0.90 | 0.34 | 0.30 | 0.59 |
|  | Caterpillar | 0.39 | 0.53 | 0.28 | 0.60 | 2.86 | 0.09 | 1.32 | 0.25 |
|  |  |  |  |  |  |  |  |  |  |
| ii) | M x Ap | 0.90 | 0.34 | 1.23 | 0.27 | 0.07 | 0.79 | 0.24 | 0.63 |
|  | M x C | 1.77 | 0.18 | 0.15 | 0.70 | 0.58 | 0.45 | 1.43 | 0.23 |
|  | Ap x C | 1.84 | 0.17 | 1.62 | 0.20 | 0.001 | 0.97 | 0.06 | 0.81 |
|  |  |  |  |  |  |  |  |  |  |
| iii) | M x date | 0.05 | 0.83 | 0.23 | 0.63 | 1.98 | 0.16 | 0.001 | 0.97 |
|  | Ap x date | 0.74 | 0.39 | 9.17 | **0.002** | 0.11 | 0.75 | 7.36 | **0.007** |
|  | C x date | 6.74 | **0.009** | 24.88 | **<0.001** | 13.52 | **<0.001** | 18.74 | **<0.001** |
|  |  |  |  |  |  |  |  |  |  |
| iv) | M x Ap x date | 18.86 | **<0.001** | 4.28 | **0.04** | 1.86 | 0.17 | 0.13 | 0.72 |
|  | M x C x date | 0.50 | 0.48 | 2.24 | 0.13 | 4.88 | **0.03** | 0.05 | 0.82 |
|  | Ap x C x date | 11.61 | **<0.001** | 20.37 | **<0.001** | 8.06 | **0.005** | 0.05 | 0.82 |
|  |  |  |  |  |  |  |  |  |  |
| v) | Acorn size | 47.18 | **<0.001** | 29.48 | **<0.001** | 30.64 | **<0.001** | 13.07 | **<0.001** |
|  | Date | 428.26 | **<0.001** | 247.56 | **<0.001** | 129.77 | **<0.001** | 326.57 | **<0.001** |
