# Supplementary material for: The timing and asymmetry of plant–pathogen–insect interactions
Source: Proc Biol Sci. 2020 Sep 23;287(1935):20201303. doi: 10.1098/rspb.2020.1303 (PMC7542815; doi:10.1098/rspb.2020.1303)
Supplement: Table S7. [file rspb20201303supp7.docx]

**Table S7**. The impact of free-feeding herbivores, aphids or both on mildew performance. Shown in panel A are the results from linear mixed models of ln-transformed mildew coverage (%) as a function of treatment, date and their interaction, with a separate model for each question (see *Table S3*). To account for repeated measures on the same plant individual, we included PlantID as a random effect. Shown in panel B are treatment- and date-specific contrasts, which were carried out using the function *emmeans* in the package *emmeans* (see *Table S4*). M = powdery mildew, Ap = aphids, C = caterpillar. N = 20 plants per treatment. Shown are degrees of freedom, test statistics and p-values.

A)

| Treatment comparisons | Treatment | | | Date | | | Treatment × Date | | |
| --- | --- | --- | --- | --- | --- | --- | --- | --- | --- |
|  | **DF** | **Χ^2^** | **p-value** | **DF** | **Χ^2^** | **p-value** | **DF** | **Χ^2^** | **p-value** |
| 2 vs. 5 vs. 9 | 2 | 18.91 | **<0.001** | 5 | 349.63 | **<0.001** | 10 | 28.78 | **0.001** |
| 2 vs. 6 vs. 11 | 2 | 4.75 | 0.09 | 5 | 416.97 | **<0.001** | 10 | 7.98 | 0.63 |
| 5 vs. 14 | 1 | 1.53 | 0.22 | 5 | 605.58 | **<0.001** | 5 | 4.90 | 0.43 |
| 6 vs. 15 | 1 | 8.77 | **0.003** | 5 | 157.80 | **<0.001** | 5 | 7.63 | 0.18 |

B)

| **Week** | **t-value** | **p-value** | **t-value** | **p-value** | **t-value** | **p-value** |
| --- | --- | --- | --- | --- | --- | --- |
|  | **2 vs. 5**  *M vs. M + Ap* | | **2 vs. 9**  *M vs. early Ap + M* | | **5 vs. 9**  *M + Ap vs. early Ap + M* | |
| Week 5 | -1.51 | 0.29 | -0.92 | 0.90 | 1.97 | 0.13 |
| Week 6 | -2.09 | 0.10 | 2.17 | 0.08 | 4.26 | **<0.001** |
| Week 7 | -2.34 | 0.06 | 2.04 | 0.11 | 4.38 | **<0.001** |
| Week 8 | -2.44 | **0.04** | 2.02 | 0.11 | 4.46 | **<0.001** |
| Week 9 | -2.43 | **0.05** | 1.98 | 0.12 | 4.41 | **<0.001** |
| Week 10 | -2.12 | 0.09 | 2.34 | 0.06 | 4.47 | **<0.001** |
|  | | | | | | |
|  | **2 vs. 6**  *M vs. M + C* | | **2 vs. 11**  *M vs. early C + M* | | **6 vs. 11**  *M + C vs. early C + M* | |
| Week 5 | -0.15 | 0.99 | -1.25 | 0.43 | -1.11 | 0.51 |
| Week 6 | 0.34 | 0.94 | -1.21 | 0.45 | -1.55 | 0.27 |
| Week 7 | -0.52 | 0.86 | -2.12 | 0.09 | -1.60 | 0.25 |
| Week 8 | -0.86 | 0.67 | -2.31 | 0.06 | -1.45 | 0.32 |
| Week 9 | -0.70 | 0.76 | -2.31 | 0.06 | -1.61 | 0.25 |
| Week 10 | -0.66 | 0.79 | -2.11 | 0.09 | -1.46 | 0.32 |
|  | | | | | | |
|  | **5 vs. 14**  *M + Ap vs. early C, then M + Ap* | | **6 vs. 15**  *M + C vs. early Ap, then M + C* | |  | |
| Week 5 | 1.05 | 0.30 | 1.08 | 0.29 |  |  |
| Week 6 | 1.71 | 0.09 | 2.36 | **0.02** |  |  |
| Week 7 | 1.51 | 0.14 | 3.12 | **0.003** |  |  |
| Week 8 | 0.87 | 0.39 | 2.91 | **0.005** |  |  |
| Week 9 | 0.95 | 0.35 | 2.55 | **0.01** |  |  |
| Week 10 | 0.93 | 0.36 | 2.89 | **0.005** |  |  |
