# Supplementary material for: The timing and asymmetry of plant–pathogen–insect interactions
Source: Proc Biol Sci. 2020 Sep 23;287(1935):20201303. doi: 10.1098/rspb.2020.1303 (PMC7542815; doi:10.1098/rspb.2020.1303)
Supplement: Table S8. [file rspb20201303supp8.docx]

**Table S8.** The impact of free-feeding herbivores, mildew or both on aphid performance. Shown in panel Aare the results from linear mixed models of ln-transformed aphid abundance as a function of treatment, date and their interaction, with a separate model for each question (see *Table S3*). To account for repeated measures on the same plant individual, we included PlantID as a random effect. Shown in panel B are treatment- and date-specific contrasts, which were carried out using the function *emmeans* in the package *emmeans* (see *Table S4*). M = powdery mildew, Ap = aphids, C = caterpillar. N = 20 plants per treatment. Shown are degrees of freedom, test statistics and p-values.

A)

| Treatment comparisons | Treatment | | | Date | | | Treatment x Date | | |
| --- | --- | --- | --- | --- | --- | --- | --- | --- | --- |
|  | **DF** | **Χ^2^** | **p-value** | **DF** | **Χ^2^** | **p-value** | **DF** | **Χ^2^** | **p-value** |
| 3 vs. 5 vs. 8 | 2 | 10.36 | **0.006** | 5 | 275.59 | **<0.001** | 10 | 31.60 | **<0.001** |
| 3 vs. 7 vs. 13 | 2 | 4.75 | 0.09 | 5 | 482.97 | **<0.001** | 10 | 8.20 | 0.61 |
| 5 vs. 14 | 1 | 6.12 | **0.01** | 5 | 142.28 | **<0.001** | 5 | 21.83 | **<0.001** |

B)

| **Week** | **t-value** | **p-value** | **t-value** | **p-value** | **t-value** | **p-value** |
| --- | --- | --- | --- | --- | --- | --- |
|  | **3 vs. 5**  *Ap vs. Ap + M* | | **3 vs. 8**  *Ap vs. early M + Ap* | | **5 vs. 8**  *Ap + M vs. early M + Ap* | |
| Week 5 | -0.12 | 0.99 | -0.92 | 0.63 | -0.80 | 0.70 |
| Week 6 | 0.80 | 0.70 | -0.12 | 0.99 | -0.92 | 0.63 |
| Week 7 | 1.55 | 0.27 | -1.10 | 0.52 | -2.65 | **0.02** |
| Week 8 | 3.05 | **0.01** | 0.00 | 1.00 | -2.84 | **0.01** |
| Week 9 | 3.10 | **0.01** | 0.26 | 0.96 | -2.84 | **0.01** |
| Week 10 | 4.13 | **<0.001** | 0.71 | 0.76 | -3.43 | **0.002** |
|  | | | | | | |
|  | **3 vs. 7**  *Ap vs. Ap + C* | | **3 vs. 13**  *Ap vs. early C + Ap* | | **7 vs. 13**  *Ap + C vs. early C + Ap* | |
| Week 5 | 0.60 | 0.82 | -1.44 | 0.32 | -2.06 | 0.10 |
| Week 6 | 0.95 | 0.61 | -1.31 | 0.39 | -2.28 | 0.06 |
| Week 7 | 0.60 | 0.82 | -1.98 | 0.12 | -2.60 | **0.03** |
| Week 8 | 0.51 | 0.87 | -1.09 | 0.52 | -1.62 | 0.24 |
| Week 9 | -0.37 | 0.93 | -1.02 | 0.57 | -0.66 | 0.79 |
| Week 10 | -0.09 | 1.00 | -0.76 | 0.73 | -0.68 | 0.77 |
|  | | | | | | |
|  | **5 vs. 14**  *Ap + M vs. early C, then Ap + M* | |  | | | |
| Week 5 | -0.19 | 0.85 |  |  |  |  |
| Week 6 | -0.38 | 0.71 |  |  |  |  |
| Week 7 | -2.00 | **0.05** |  |  |  |  |
| Week 8 | -3.18 | **0.002** |  |  |  |  |
| Week 9 | -2.94 | **0.004** |  |  |  |  |
| Week 10 | -3.16 | **0.002** |  |  |  |  |
