# Supplementary material for: The timing and asymmetry of plant–pathogen–insect interactions
Source: Proc Biol Sci. 2020 Sep 23;287(1935):20201303. doi: 10.1098/rspb.2020.1303 (PMC7542815; doi:10.1098/rspb.2020.1303)
Supplement: Figure S1. [file rspb20201303supp9.docx]

**Figure S1.** The impact of treatment on plant performance through time. Shown are data for A) plant height, B) number of developed leaves, C) leaf size and D) number of shoots from week 4 to 10. See Fig. 1 for an overview of the fifteen treatments. N = 300 plants, with 20 plants per treatment. Error bars represent standard errors.


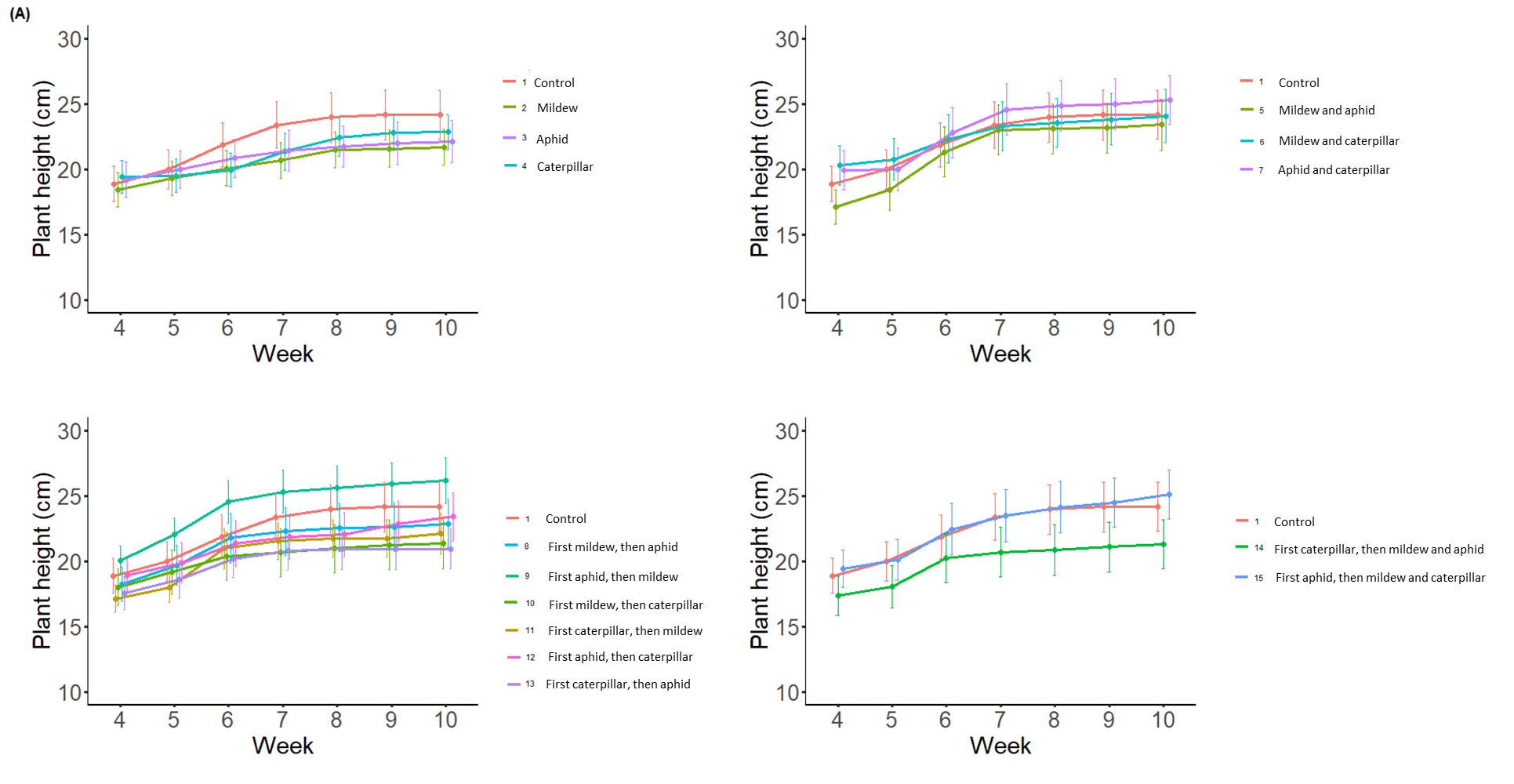


**
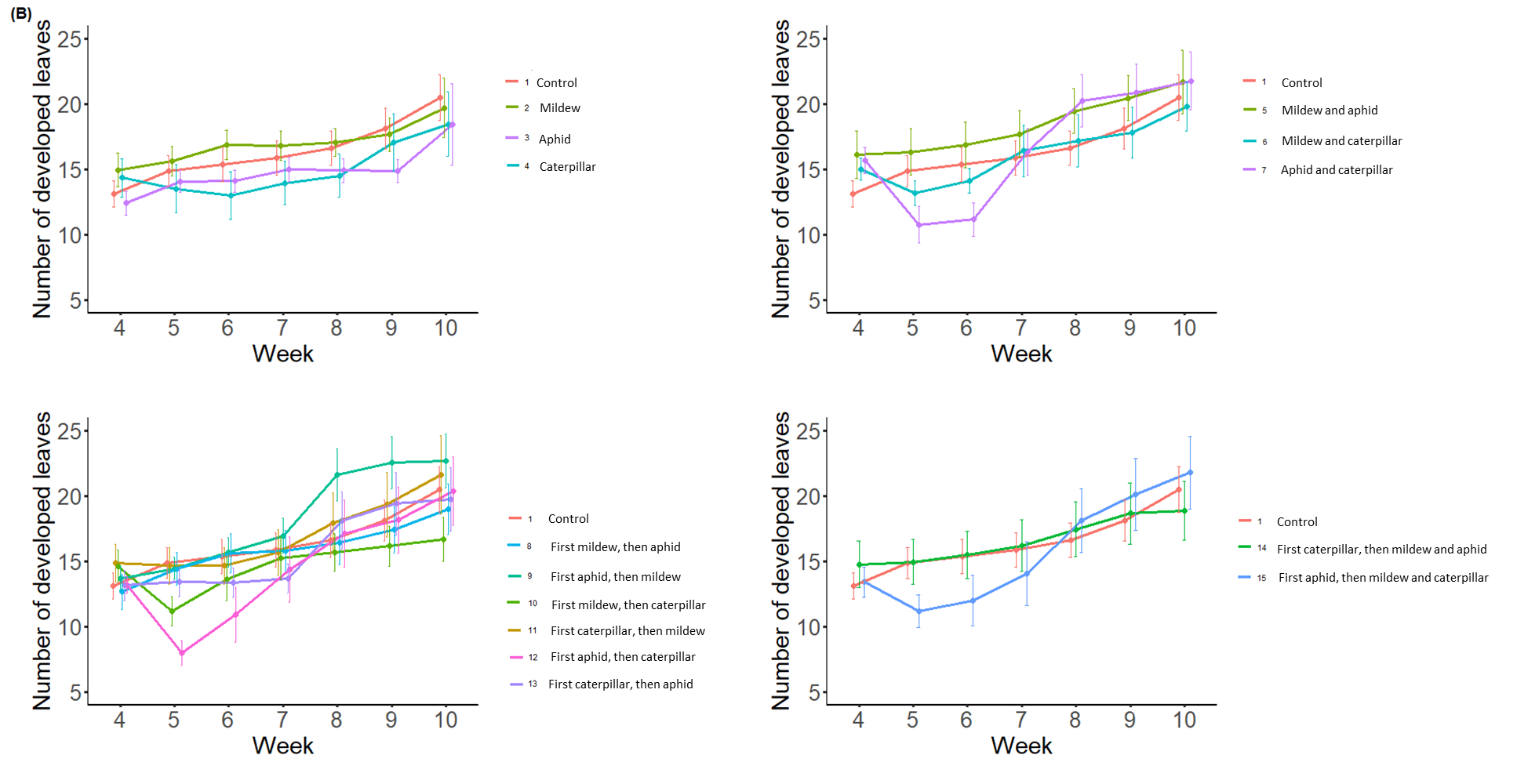

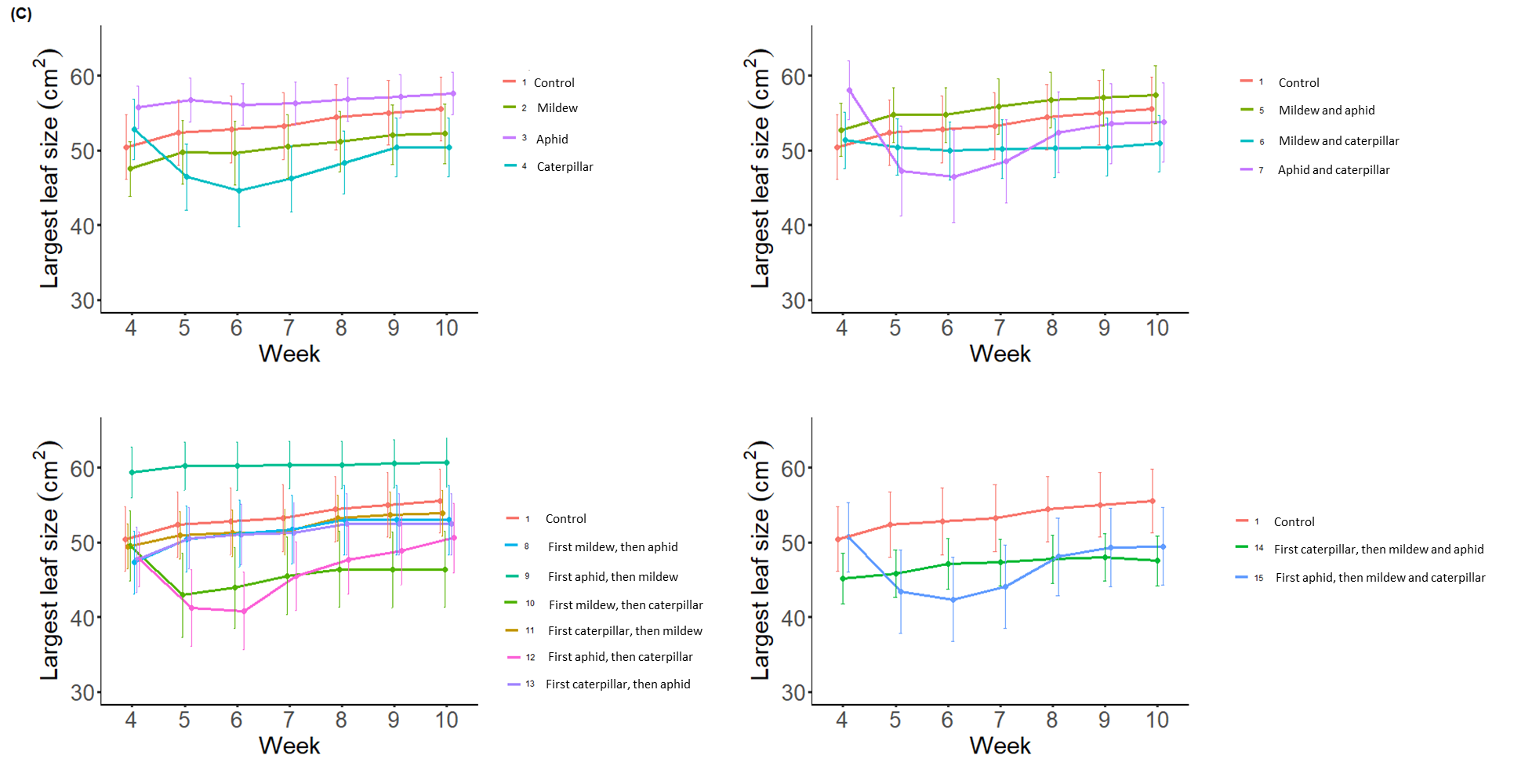
**

**
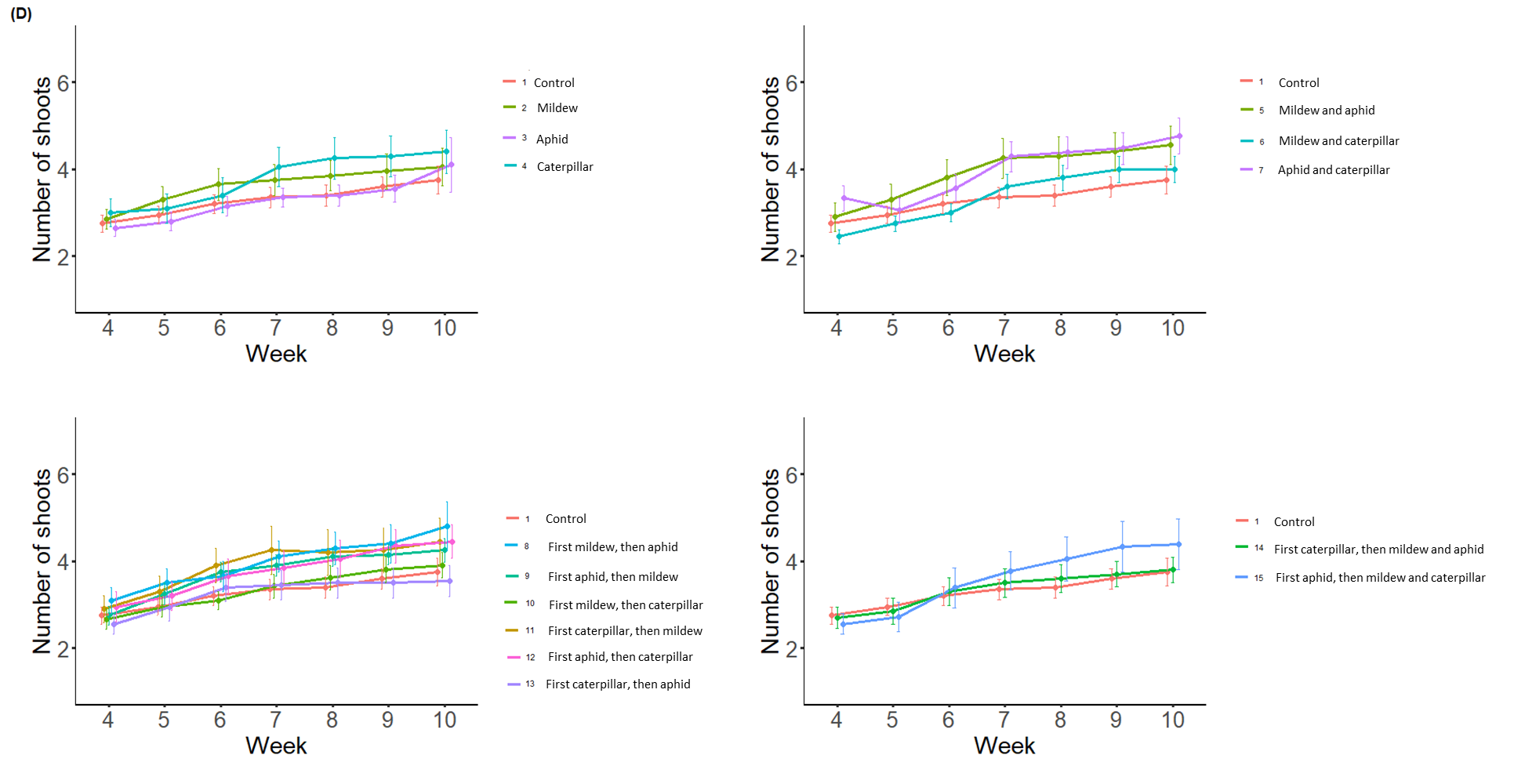
**
