# Supplementary material for: The timing and asymmetry of plant–pathogen–insect interactions
Source: Proc Biol Sci. 2020 Sep 23;287(1935):20201303. doi: 10.1098/rspb.2020.1303 (PMC7542815; doi:10.1098/rspb.2020.1303)

**Figure S3.** The impact of single and dual attack on plant performance through time. Panels A-D show the impact of single attackers (mildew, aphids and caterpillars) on plant height, number of developed leaves, leaf size and number of shoots from week 4 to 10. Panels E-H show examples of variable impact of dual attack on plant performance through time. Error bars represent standard errors. Asterisks indicate a significant difference with the control treatment or a significant interaction. * = p-value < 0.05, ** = p-value < 0.01.

**
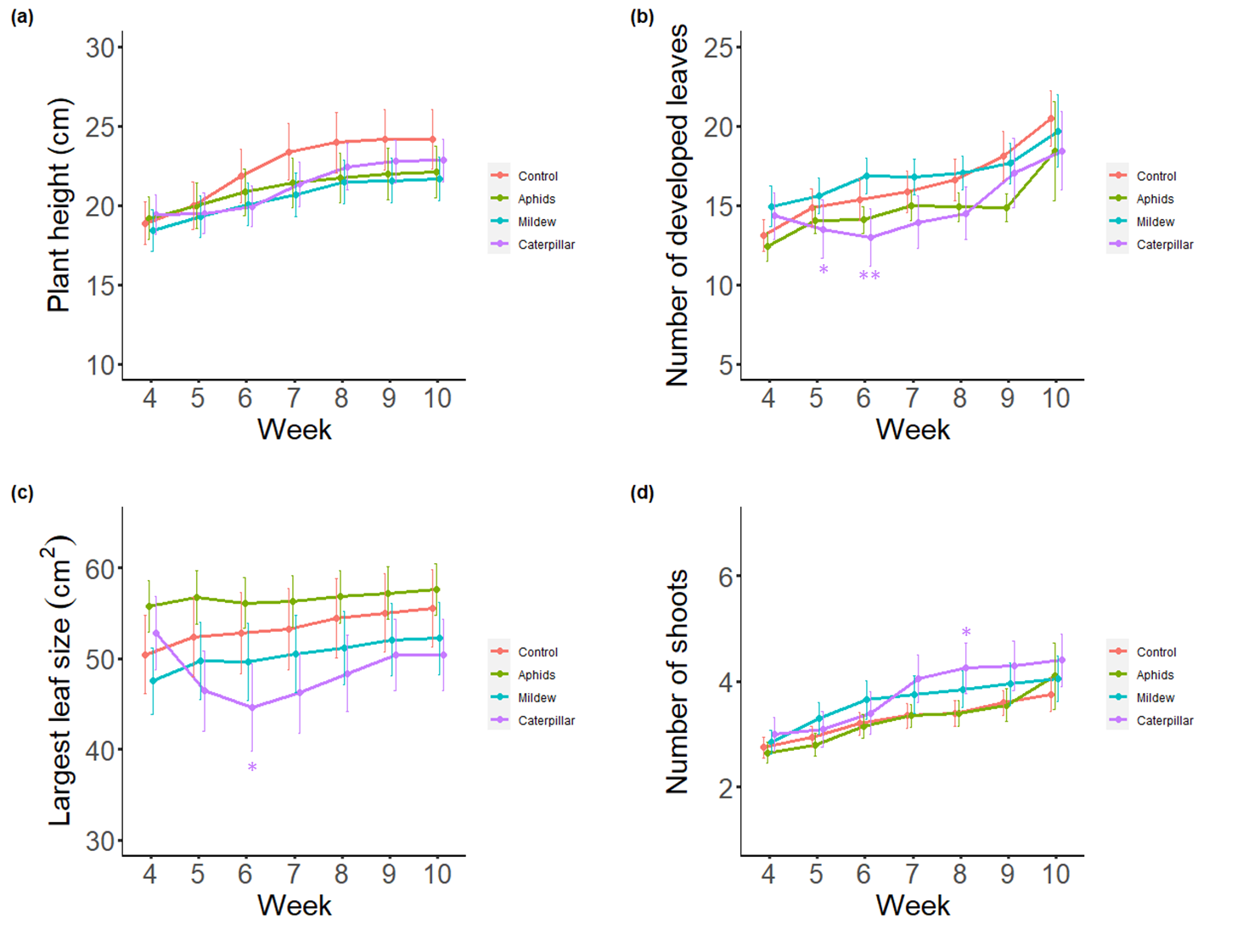
**


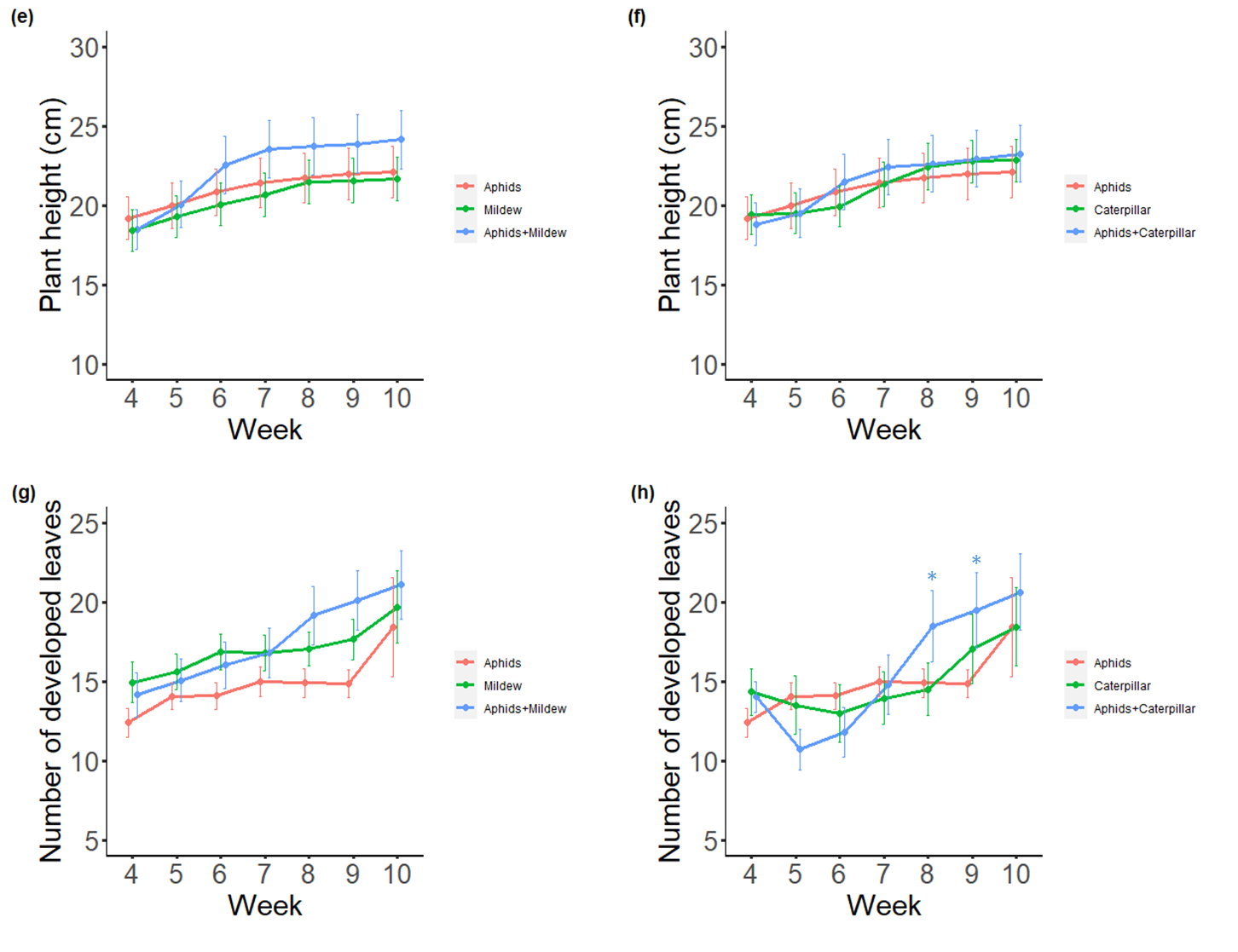

Supplement: Figure S3. [file rspb20201303supp10.docx]
