# Supplementary material for: The timing and asymmetry of plant–pathogen–insect interactions
Source: Proc Biol Sci. 2020 Sep 23;287(1935):20201303. doi: 10.1098/rspb.2020.1303 (PMC7542815; doi:10.1098/rspb.2020.1303)

**Figure S2.** The impact of acorn size and attackers on plant height. A: The impact of acorn size (category 1 – 6) on plant height in week 10. B: The impact of attackers, and combinations of attackers, on plant height in week 10. Error bars represent standard errors. M = Mildew, Ap = Aphids, C = Caterpillar.


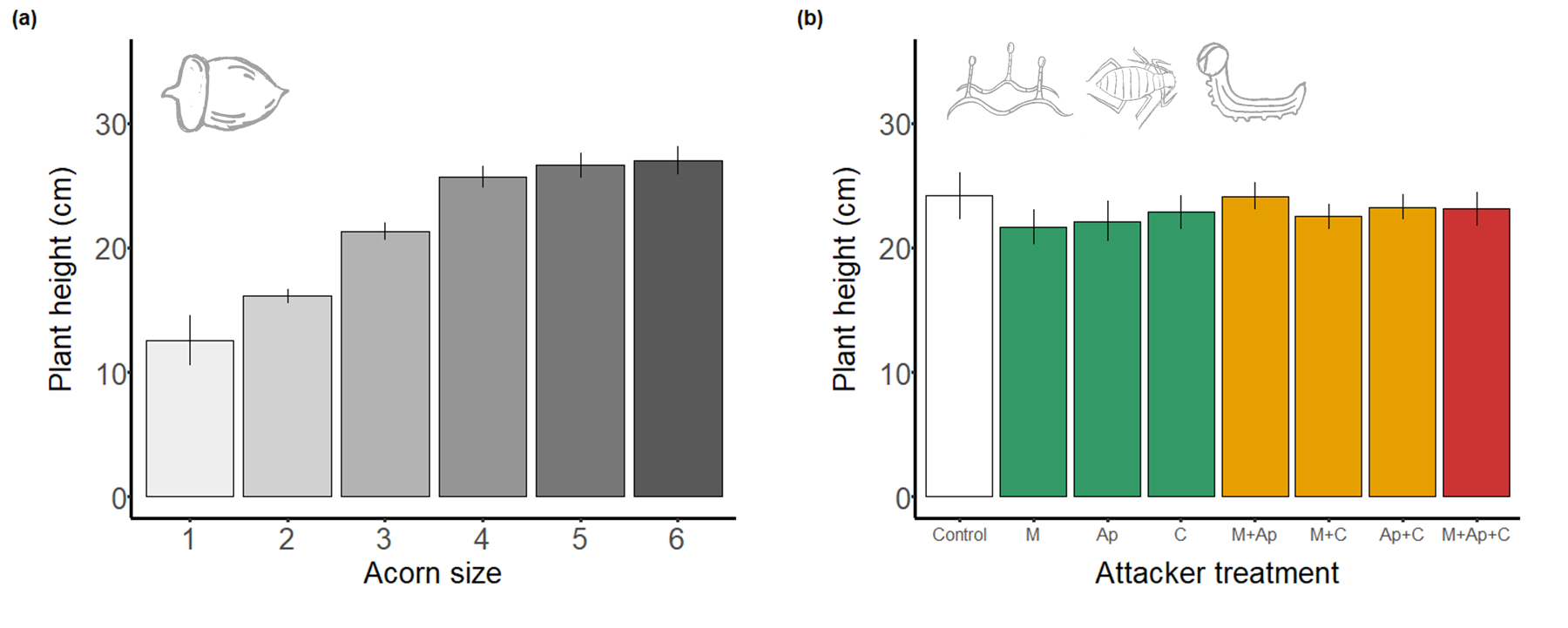

Supplement: Figure S2. [file rspb20201303supp11.docx]
