# Supplementary material for: The timing and asymmetry of plant–pathogen–insect interactions
Source: Proc Biol Sci. 2020 Sep 23;287(1935):20201303. doi: 10.1098/rspb.2020.1303 (PMC7542815; doi:10.1098/rspb.2020.1303)

**Figure S4**. The effect of aphids and mildew on caterpillar performance. A: The mean caterpillar growth rate when the caterpillar is alone on a plant (4), when the caterpillar is co-occurring with aphids (7) and when aphids arrive earlier than the caterpillar (12). Caterpillar growth rate was calculated as the caterpillar weight after feeding on an aphid-infested plant for one week, divided by the starting weight at the beginning of the week. Each treatment had 20 replicates. B: Caterpillar survival rate when feeding on mildew infected leaves versus healthy leaves. N = 130 caterpillars on a healthy diet, and 81 caterpillars on a mildew diet. C: The mean caterpillar development time in weeks when feeding on healthy leaves versus mildew infected leaves. Error bars present the standard errors.


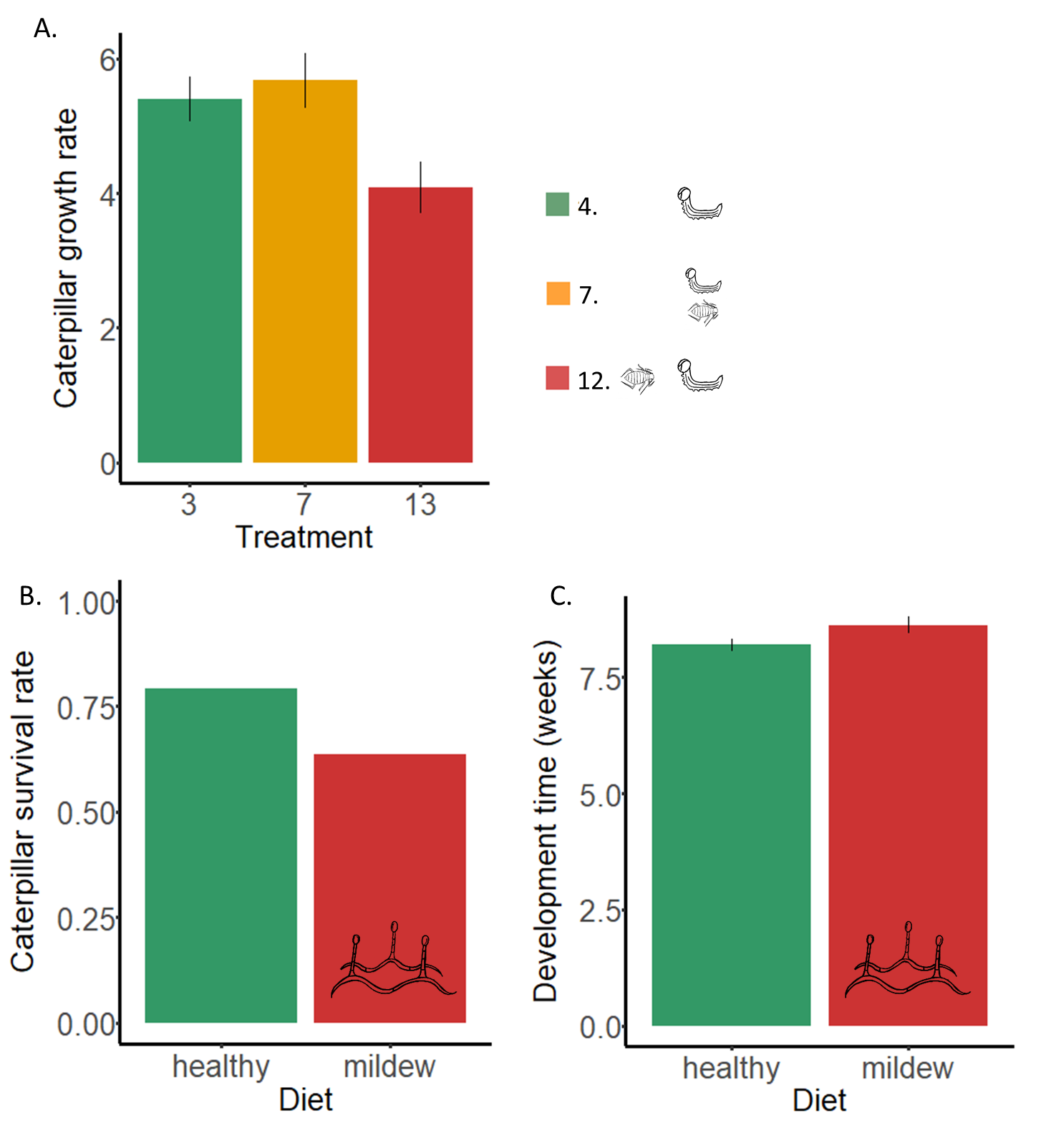

Supplement: Figure S4. [file rspb20201303supp12.docx]
